# Waist circumference trajectories and risk of type 2 diabetes mellitus in Korean population: the Korean genome and epidemiology study (KoGES)

**DOI:** 10.1186/s12889-019-7077-6

**Published:** 2019-06-13

**Authors:** Jooeun Jeon, Keum Ji Jung, Sun Ha Jee

**Affiliations:** 1 Department of Public Health, Graduate School, Yonsei University, 50 Yonsei-ro, Seodaemun-gu, Seoul, 03722 Republic of Korea; 2 Department of Epidemiology and Health Promotion, Institute for Health Promotion, Graduate School of Public Health, Yonsei University, 50 Yonsei-ro, Seodaemun-gu, Seoul, 03722 Republic of Korea

**Keywords:** Waist circumference, Type 2 diabetes mellitus, Longitudinal study, Trajectory

## Abstract

**Background:**

To classify waist circumference (WC) trajectories and examine each trajectory’s association with risk of incident type 2 diabetes mellitus (T2DM).

**Methods:**

In Korean Genome and Epidemiology Study (KoGES 2001–2014), 4992 participants aged 40 years and above who received biennial health examinations from wave 1 to wave 4 (2001–2008) were selected. Five distinct trajectory groups were identified for WC using group-based trajectory modeling methods such as censored normal model. Cox proportional hazards model was used to examine the association of trajectories with risk of T2DM.

**Results:**

During 31,118 person-years of follow-up (mean follow-up duration, 6.2 years), 276 incident cases of T2DM were identified. Through trajectory analysis, 5 distinct WC patterns were found during wave1 to wave 4, which were “Group A” was stable on very low levels, “Group B” was stable on low levels, “Group C” was stable on moderate levels, “Group D” had increasing pattern on elevated levels, “Group E” was shown increasing on high levels. Age-standardized incidences rates per 100,000 person-years were increased with WC expanding trajectory group (193.9 for Group A, 498.4 for Group B, 661.9 for Group C, 1845.9 for Group D, and 2045.0 for Group E). In multivariate analysis after adjusting for confounding variable at wave 4, Group B (Hazard ratio (HR), 2.2; 95% confidence interval (CI), and 1.1–4.6), Group C (HR: 2.5, 95% CI: 1.2–5.0), Group D (HR: 5.4, 95% CI: 2.7–10.9), Group E (HR: 7.3, 95% CI: 3.5–15.4) had a higher risk of T2DM than Group A. After further adjusting for body mass index strongly correlated with WC, the association was attenuated.

**Conclusions:**

WC trajectory was a significant predictor of T2DM risk in increasing trajectories on high level. This finding indicate the importance of WC management across prolong lifespan by assessing the prognosis and prevention strategies of high-risk populations for T2DM in middle-aged adults.

**Electronic supplementary material:**

The online version of this article (10.1186/s12889-019-7077-6) contains supplementary material, which is available to authorized users.

## Background

Type 2 diabetes mellitus (T2DM) is the most common metabolic disorder in the world. Mortality and morbidity of T2DM are consistently increasing [1, 2]. Both T2DM and prediabetes risk defined by impaired fasting glucose are increasing constantly in early adulthood with obesity [3]. Meanwhile, obese population in the world is increasing rapidly [4]. In previous multiethnic cohort study, overweight, excess energy intake, and physical inactivity have been associated with increased number of diabetes patients [5]. According to study of obesity by Korean society, the rate of obesity in Korean adults is 32.4%, with one-third of them being obese recently [6]. In recent decades, extreme obesity rate has increased by 78% and abdominal obesity in those aged 20–40 years among Koreans has increased rapidly [6]. Obese Korean adults have shown a more than 2-fold of increase in the risk of T2DM compared to non-obese subjects [7]. Indeed, in a previous cohort study, elevated risk of cardiovascular disease prior to clinical diagnosis of T2DM has been reported [8]. For this reason, managing risk factors related to cardiovascular disease in middle adulthood and helping adults, living in the community to notice early signs of T2DM and detect T2DM in its early stages has significant public health implications [9]. However, T2DM recognition and treatment rates for Korean adults aged 30–40 years are very low [7]. In addition, many young adults in Korea do not habitually engage in regular medical examinations. As a result, the diagnosis of T2DM by screenings is delayed to middle-adult. Meanwhile, Asian obesity have lean shape and were healthier than Western. So, previous related studies have suggested that waist circumference (WC) may be a better predictor of T2DM than body mass index (BMI) in Asian adults [10, 11]. And, BMI may not be a direct measure of body fat because it does not reflect gender differences in body fat loads [11]. Abdominal obesity is the single-most important risk factor for metabolic disorder and its predisposition to T2DM [12]. WC is a simple and reliable anthropometric measure used in epidemiological studies as a substitute for central obesity [13]. However, WC captures an estimate of subcutaneous abdominal fat tissue as well as the intra-abdominal fat tissue. These two compartments are metabolically active and contribute significantly to metabolic dysfunction, including insulin resistance and T2DM [14].

In recent, it is possible to determine changes of anthropometric measurements (such as weight, height, waist and hip circumference) pattern based on different aspects of their over time. In addition, effects of distinct pattern of WC trajectories on actual appearance of T2DM are visible depending on group-based trajectory modeling method [15, 16].

Therefore, the aims of the present study were: 1) to apply trajectory models to identify distinct trajectories of WC in Korean adults aged 40 years or more at baseline; and 2) to examine the association between trajectories of WC and T2DM incidence.

## Methods

### Participants

Data were obtained from Korean genome and epidemiology study (KoGES), a 7-wave longitudinal data that included 245,000 Korean adults aged 40 years or more based on baseline (2001–2002) from 2001 to 2014 [17]. KoGES Ansan and Ansung study (one subset of KoGES in which biannual repeated surveys were continued since baseline recruitment in 2001–2002 up to the 6th follow-up) were used. Out of 10,030 baseline participants, the 6th follow-up was conducted in 5906 participants out of 9397 survivors. Approximately 90% of baseline participants completed at least one follow-up survey over the course of 12 years [17]. The study protocol was approved by the Ethics Committee of the Korean Center for Disease Control, the Institutional Review Boards of the Korea University Ansan Hospital and the Ajou University School of Medicine. From both KoGES (Ansung-Ansan) cohort study, 6133 individuals who received biennial health evaluation routinely from the baseline to the 6th follow-up were included.

### Ascertainment of T2DM and follow-up

**Fig. 1:**
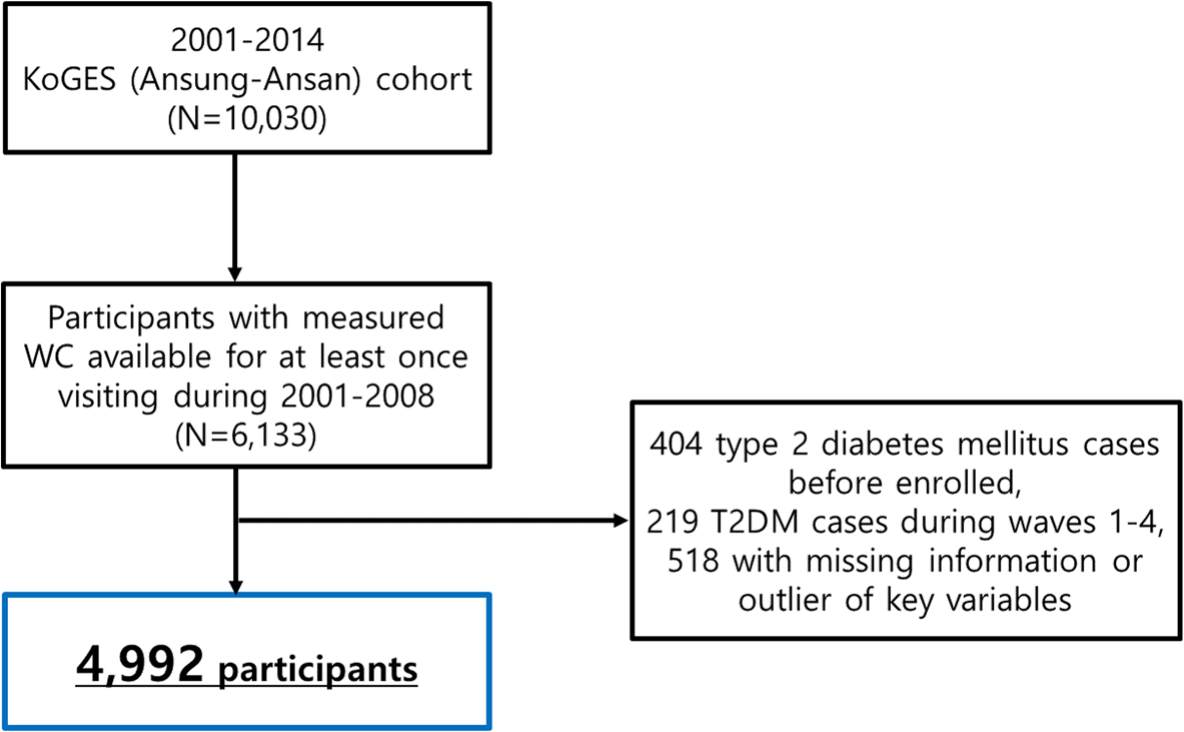
Flow chart for the selection of the study population

T2DM was defined based on a self-reported current treatment with anti-diabetes medication and/or as fasting glucose ≥126 mg/dl and/or glycated hemoglobin (HbA1c) ≥6.5% by KoGES (Ansung-Ansan) cohort’s questionnaire. For more detailed information of KoGES (Ansung-Ansan) cohort’s questionnaire, we could confirm through previous cohort profile publication for this cohort [17]. Among 6133 individuals, 404 individuals with diagnosis of T2DM at the baseline, 219 individuals who had T2DM during waves 1–4 (2001–2008), and 518 individuals with missing or outlier values of key variables were excluded. Finally, 4992 participants (2342 men and 2650 women) were included in the final sample (Fig. 1). Follow-ups for all participants were conducted from wave 4 to December 31, 2014. For T2DM cases whose dates of T2DM diagnosis (year, month, and day) could not be ascertained, T2DM was defined autonomously because of data attribute. Follow-ups for T2DM cases were conducted from wave 4 to onset of T2DM. Finally, 276 cases of T2DM were occurred during the follow-up (1st to 6th).

### Measures of waist circumference and covariates

Anthropometric measurements were also obtained (i.e. height, weight, waist circumference) by trained research staff in KoGES (Ansung-Ansan) cohort study. The WC unit was centimeter and each WC levels during wave 1–4 were used to trajectory analysis. BMI was calculated as weight (in kilograms) divided by square of height (in meters).

The following self-reported variables were used as categorical variables: smoking status (non-smoker, ex-smoker, and current-smoker), drinking status (non-drinker, ex-drinker, and current-drinker), and regular exercise (regular exercise, and no exercise). Physical and laboratory examinations were conducted to collect clinical variables. Age, sex, weight, height, waist circumference, systolic blood pressure, total cholesterol, fasting blood sugar, smoking status, drinking status, and regular exercise at wave 4 were incorporated as covariates in the present study.

Other variables in the main questionnaire of KoGES (Ansung-Ansan) cohort not used in this study are detailed in the Korean Centers for Disease Control and Prevention (KCDC) site (http://www.nih.go.kr/NIH/eng/contents/NihEngContentView.jsp?cid=65199&menuIds=HOME004-MNU2261-MNU2262-MNU2263-MNU2264).

### Statistical analysis

**Fig. 2:**
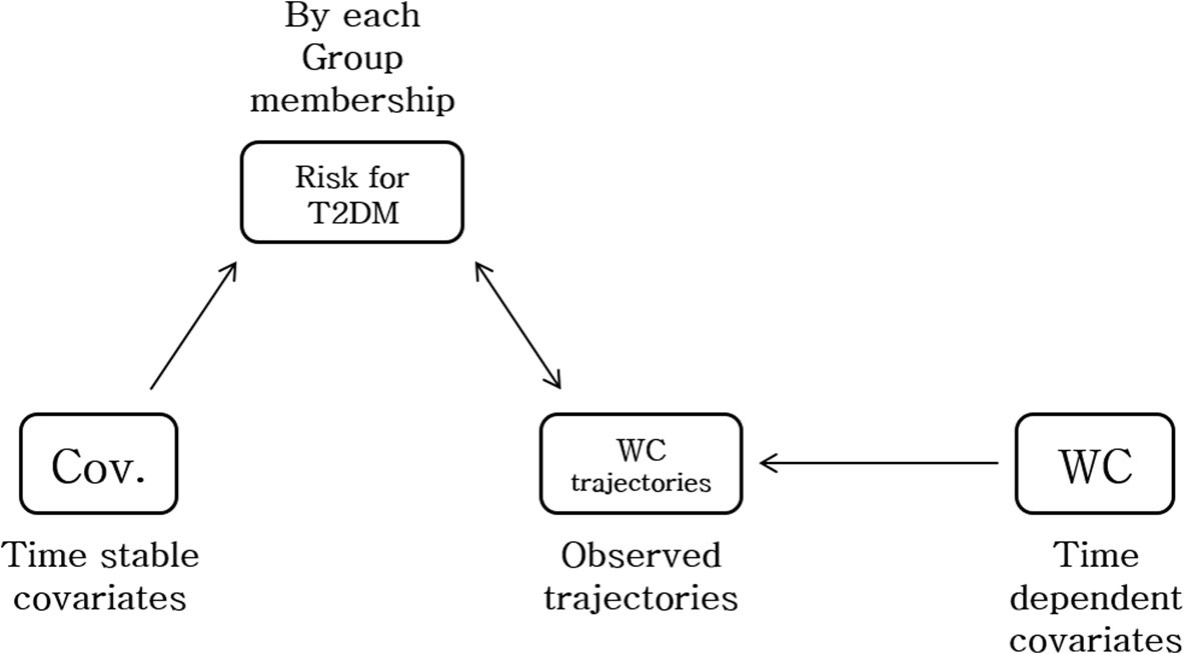
Basic assumption of group-based trajectory modeling methods in this study. Abbreviation: T2DM, type 2 diabetes mellitus; WC, waist circumference

A group-based trajectory model (GBTM) was used with a nonparametric maximum likelihood estimation to identify a clear trajectory of WC changes during follow-up until the first T2DM event occurred [15, 18]. In this study, latent class growth modeling (LCGM) was performed using one of the GBTM. The longitudinal nature of data has been modeled by having parameters over time. Time-stable covariates (risk factors) are incorporated into the model assuming that they can affect the probability of belonging to a particular group. As shown in Fig. 2, covariance over time can also directly affect the observed patterns [9]. BMI have strong correlation to WC was included as time dependent covariate related to WC. It was assumed that all subjects in the study sample were from a single population. Therefore, one (average) trajectory should adequately describe the developmental pattern of the sample [19].

**Fig. 3:**
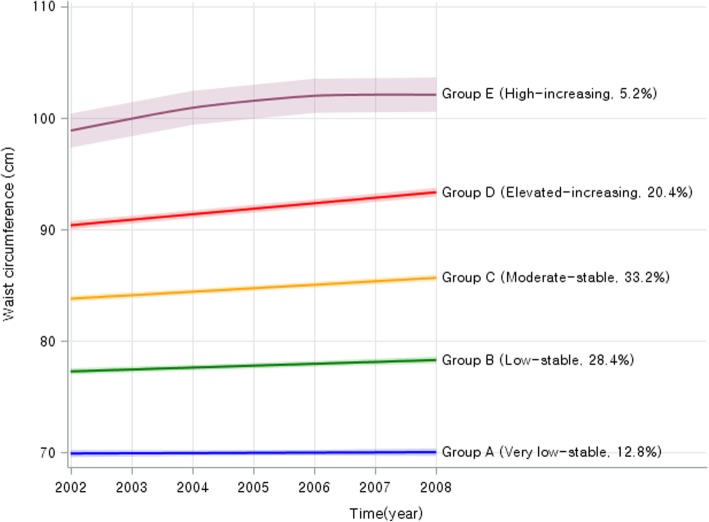
Changes in waist circumference during waves 1–4 by trajectory groups

This analysis identified a relatively homogeneous cluster of trajectories of change over time in repeated observations on the subject of analysis using multidimensional mixed modeling strategies [20]. As recommended, the optimal fit model is based on the BIC indices associated with automatic calculations and the average posterior probability of group members of each latent class for each participant (> 0.7) as well as clinical plausibility [21]. To obtain the best model with the number of distinct trajectories and satisfying several assumptions, we estimated the appropriate WC trajectory model by increasing the number of clusters from three to seven under assuming a linear, quadratic and tertiary pattern variation of WC over time by SAS PROC TRAJ package. While estimating the appropriate trajectory model, we putted BMI values during wave 1–4 as the risk variables of model in the model to consider significant correlation between WC and BMI (Additional file 2: Figure S2). Each participant was assigned to each class with the highest probability. To ensure that all acquired classes are of clinically meaningful size, the condition was imposed that each class should include at least 5% of the participants and that the average rearward probability of each class should be at least 75% [18].

Finally, five distinct WC trajectories were identified (Fig. 3). After obtaining trajectories, within each identified WC group, trajectories of change in other related risk factors during the follow-up were developed and independent effects of WC trajectories on T2DM risk by person years per 100,000 were assessed. Subsequently, after evaluate the proportional hazards assumption, multivariate Cox proportional hazard model was fitted after adjusting for covariates at wave 4 to quantify the association between BMI trajectory groups and T2DM risk. We also estimated interaction effect for T2DM risk between WC trajectories and gender using by the multivariate Cox proportional hazard model added type 3 option with LR commend, which can test based on a likelihood ratio test to calculate *P* values for interaction. Further analyses were conducted after stratified by gender and smoking status (non-smoker or ever smoker). We also additionally conducted gender-specific trajectory analyses to compare with results from stratified model. All analyses were performed using SAS statistical software version 9.4 (Institute, Inc., Cary, North Carolina, USA). All other statistical tests were two-sided and statistical significance was determined as *p* < 0.05.

## Results

Of 4992 participants, women had a higher proportion (53.1%) than men. Half of all participants were less than 50 years old in this study. Overall, the average BMI range for study subjects was stable at 24.5 kg/m^2^ during the follow-up until wave 4 in both of gender. Also, the mean value of WC was 82.1 cm at wave 1 and 83.7 at wave 4. Men had a higher mean value of WC (85.3 cm) compared to women with a mean value of WC of 82.4 cm at wave 4 (baseline for estimation risk of T2DM).

As a result of group-based trajectory modeling analysis, the model with five trajectories had a better fit than that with other number of trajectories. Probability for each group was statistically significant (*p* < 0.0001). BIC scores for the number of groups were − 60,001.72. General characteristics of these five BMI trajectory groups are summarized in Table 1. WC trajectories patterns derived from group-based trajectory models are shown in Fig. 1. In result from trajectory analysis, there were 5 group during wave1 to wave 4, which were “Group A” was stable on very low levels, “Group B” was stable on low levels, “Group C” was stable on moderate levels, “Group D” had increasing pattern on elevated levels, “Group E” was shown increasing on high levels. The following five distinct trajectories were identified: “Group A (Very low-stable)” (baseline WC = 69.9, standard deviation (SD) = 4.5; wave 4 BMI = 69.5, SD = 4.4); “Group B (Low-stable)” (baseline WC = 77.1, SD = 4.4; wave 4 WC = 77.8, SD = 4.2); “Group C (Moderate-stable)” (baseline WC = 83.7, SD = 4.0; wave 4 WC = 85.5, SD = 4.0); “Group D (Elevated-increasing)” (baseline WC = 90.1, SD = 4.0; wave 4 WC = 93.3, SD = 3.9); and “Group E (High-increasing)” (baseline WC = 98.8, SD = 5.0; wave 4 WC = 102.3, SD = 4.7). We could not only identify the changed patterns for group D and E, but also identify significance increasing WC mean value for both of groups from trajectory analysis (Additional file 1: Table S1).

**Table 1:** Basic characteristics by waist circumference trajectories at baseline and wave 4 (*N* = 4992)

	Waist circumference (WC) trajectory					*p*-value
	Group A (Very low-stable)	Group B (Low-stable)	Group C (Moderate-stable)	Group D (Elevated-increasing)	Group E (High-increasing)	
*N* = 4992	*N* = 635	*N* = 1426	*N* = 1662	*N* = 1012	*N* = 257	
Waist circumference, cm						
at baseline	69.9 ± 4.5	77.1 ± 4.4	83.7 ± 4.0	90.1 ± 4.0	98.8 ± 5.0	<.0001
at wave 4	69.5 ± 4.4	77.8 ± 4.2	85.5 ± 4.0	93.3 ± 3.9	102.3 ± 4.7	<.0001
BMI, kg/m^2^						
at baseline	21.0 ± 2.0	23.1 ± 2.1	24.7 ± 2.0	26.7 ± 2.1	29.9 ± 2.5	<.0001
at wave 4	20.9 ± 2.0	22.9 ± 2.0	24.6 ± 1.9	26.7 ± 2.1	29.8 ± 2.6	<.0001
Fasting glucose, mg/dl						
at baseline	84.7 ± 7.7	86.5 ± 8.0	87.7 ± 8.2	88.6 ± 8.1	88.9 ± 8.4	<.0001
at wave 4	86.8 ± 8.0	90.5 ± 8.3	93.3 ± 9.1	95.7 ± 9.8	95.4 ± 9.7	<.0001
Systolic BP, mmHg						
at baseline	110.4 ± 17.3	115.6 ± 16.8	120.8 ± 17.5	125.9 ± 17.5	129.9 ± 18.4	<.0001
at wave 4	107.4 ± 15.6	113.3 ± 15.0	118.7 ± 15.1	123.4 ± 15.6	127.3 ± 15.3	<.0001
Diastolic BP, mmHg						
at baseline	77.6 ± 11.1	77.3 ± 10.7	81.3 ± 11.3	84.2 ± 11.1	86.6 ± 10.9	<.0001
at wave 4	74.7 ± 9.5	74.8 ± 9.5	78.3 ± 9.4	81.1 ± 9.1	82.5 ± 8.9	<.0001
Total cholesterol, mg/dl						
at baseline	188.8 ± 32.7	192.6 ± 34.4	198.5 ± 35.0	201.8 ± 33.5	206.3 ± 38.2	<.0001
at wave 4	194.9 ± 34.1	195.6 ± 34.9	196.3 ± 34.3	196.6 ± 34.5	198.6 ± 31.4	0.6138
*N* (%)						
Age, years						
< 50	235 (37.0)	375 (26.3)	344 (20.7)	133 (13.1)	28 (10.9)	<.0001
50–59	246 (38.7)	635 (44.5)	658 (39.6)	386 (38.1)	71 (27.6)	
60+	154 (24.3)	416 (29.2)	660 (39.7)	493 (48.7)	158 (61.5)	
Sex						
Women	463 (72.9)	809 (56.7)	716 (43.1)	488 (48.2)	174 (67.7)	<.0001
Smoking status						
Former smoker	63 (9.9)	221 (15.5)	416 (25.0)	235 (23.2)	34 (13.2)	<.0001
Current smoker	74 (11.7)	237 (16.6)	307 (18.5)	166 (16.4)	30 (11.7)	
Drinking status						
Yes	226 (35.6)	669 (46.9)	900 (54.2)	472 (46.6)	108 (42.0)	<.0001
Regular exercise						
Yes	294 (46.3)	624 (43.8)	677 (40.7)	362 (35.8)	74 (28.8)	<.0001

Abbreviation: *BMI* body mass index, *BP* blood pressure, *SD* standard deviation

Results for the risk of T2DM hazard ratio for each WC trajectory group with “Group A” trajectory as a reference are shown in Table 2. During 31,118 person-years of follow-up (mean follow-up, 6.2 years), 276 incident cases of T2DM were documented. Age-standardized incidence rates per 100,000 person years were increased with WC levels: 193.9 in the Group A, 498.4 in the Group B, 661.9 in the Group C, 1845.9 in the Group D, and 2045.0 in the Group E. In multivariate Cox proportional hazard model after adjusting for age, age square, sex and family history for T2DM, the “Group B (Low-stable)” had a significantly higher risk of T2DM (hazard ratio (HR): 2.6, 95% confidence interval (CI): 1.3–5.2) compared to Group A. All other trajectories “Group D (Elevated-increasing)”, “Group D (Elevated-increasing)”, and “Group E (High-increasing)” also had significantly higher risk of T2DM (HR: 3.2, 95% CI: 1.6–6.4; HR: 7.4, 95% CI: 3.7–14.8; HR: 10.0, 95% CI: 4.8–20.7, respectively) compared to the Group A. In model 3 after adjusting for health behaviors and biological indicators of subclinical illness (smoking status, alcohol intake, physical exercise, systolic blood pressure, and total cholesterol at wave 4) additionally, Group B (Hazard ratio (HR), 2.2; 95% confidence interval (CI), and 1.1–4.6), Group C (HR: 2.5, 95% CI: 1.2–5.0), Group D (HR: 5.4, 95% CI: 2.7–10.9), Group E (HR: 7.3, 95% CI: 3.5–15.4) were significantly associated with T2DM risk. After added BMI as a covariate in the model to confirm whether did well adjust BMI in trajectory analysis or not, the risk of T2DM were shown more attenuated by each trajectories than before (data was not shown).

**Table 2:** Hazard ratios (95%CI) for risk of type 2 diabetes according to waist circumference group by trajectory analysis, *N* = 4, 992

	No. of Persons	No. of T2DM incidences	Person years, follow-up	Age adjusted rate	Model 1^a^	Model 2^b^	Model 3^c^
					HR (95% CI)	HR (95% CI)	HR (95% CI)
WC Group A	635	9	4033.6	193.9	1.0	1.0	1.0
WC Group B	1426	52	8984.2	498.4	2.6 (1.3–5.2)	2.5 (1.2–5.0)	2.2 (1.1–4.6)
WC Group C	1662	75	10,392.1	661.9	3.2 (1.6–6.4)	3.0 (1.5–5.9)	2.5 (1.2–5.0)
WC Group D	1012	104	6158.6	1845.9	7.4 (3.7–14.8)	7.0 (3.5–13.8)	5.4 (2.7–10.9)
WC Group E	257	36	1549.2	2045.0	10.0 (4.8–20.7)	10.0 (4.8–20.8)	7.3 (3.5–15.4)
P for trend					<.0001	<.0001	<.0001
C statistic					0.6841	0.6956	0.8689

Abbreviation: *T2DM* type 2 diabetes mellitus, *WC* waist circumference, *HR* hazard ratio, *CI* confidence interval

^a^Model 1: Adjusted for age, age square, sex, and family history of type 2 diabetes mellitus at wave 4

^b^Model 2: Adjusted for Model 2 + smoking status, alcohol intake, physical exercise, and body mass index at wave 4

^c^Model 3: Adjusted for Model 3 + systolic blood pressure, and total cholesterol at wave 4

Stratifying analyses by sex or smoking status was conducted to compare findings by rerunning all models. Results of stratified by sex are shown in Table 3. These results addressed different aspects by sex for the risk of T2DM. After adjusting for demographics, health behaviors, biological indicators of subclinical illness, and family history for T2DM at wave 3, “Group D (Elevated-increasing)” (HR: 3.7, 95% CI: 1.3–10.4) and “Group E (High-increasing)” (HR: 7.8, 95% CI: 2.6–23.5) had significantly higher risk of T2DM compared to “Group A” in men. In women, all trajectories had significantly higher risk of T2DM (HR: 3.2, 95% CI: 1.3–8.3 for Group B (Low-stable); HR: 3.1, 95% CI: 1.2–8.0 for Group C (Moderate-stable); HR: 6.5, 95% CI: 2.5–16.9 for Group D (Elevated-increasing), HR: 6.5, 95% CI: 2.3–18.1 for Group E (High-increasing), respectively) compared to “Group A” trajectory in women. After adjusting for BMI additionally, no trajectory had significantly higher risk of T2DM. There was no significant interaction in gender by WC trajectories (P for interaction**: 0.1262**).

**Table 3:** Hazard ratio with 95% confidence interval for risk of type 2 diabetes according to waist circumference trajectory group by gender (*N* = 4, 992)

	Men (*N* = 2342)			Women (*N* = 2650)		
	Model1^a^	Model2^b^	Model3^c^	Model1^a^	Model2^b^	Model3^c^
	HR (95% CI)	HR (95% CI)	HR (95% CI)	HR (95% CI)	HR (95% CI)	HR (95% CI)
WC Group A	1.0	1.0	1.0	1.0	1.0	1.0
WC Group B	1.4 (0.5–4.0)	1.3 (0.5–3.9)	1.2 (0.4–3.6)	3.5 (1.4–9.0)	3.5 (1.4–9.0)	3.2 (1.3–8.3)
WC Group C	1.9 (0.7–5.4)	1.9 (0.7–5.4)	1.7 (0.6–4.8)	3.6 (1.4–9.3)	3.7 (1.4–9.6)	3.1 (1.2–8.0)
WC Group D	4.5 (1.6–12.5)	4.4 (1.6–12.3)	3.7 (1.3–10.4)	8.3 (3.2–21.3)	8.6 (3.4–22.2)	6.5 (2.5–16.9)
WC Group E	9.9 (3.3–29.3)	10.0 (3.4–29.5)	7.8 (2.6–23.5)	8.5 (3.1–23.3)	8.8 (3.2–24.4)	6.4 (2.3–18.1)
P for trend	<.0001	<.0001	<.0001	<.0001	<.0001	<.0001
P for interaction	0.1262*					

Abbreviation: *WC* waist circumference, *HR* hazard ratio, *CI* confidence interval; * there was no interaction between WC trajectories and gender

^a^Model 1: Adjusted for age, age square, and family history of type 2 diabetes mellitus at wave 4

^b^Model 2: Adjusted for Model 2 + smoking status, alcohol intake, physical exercise, and body mass index at wave 4

^c^Model 3: Adjusted for Model 3 + systolic blood pressure, and total cholesterol at wave 4

Table 4 was shown hazard ratio for risk of T2DM by smoking status (ever smokers, never smokers). We could find that non-smoker had significantly high risk for T2DM (HR: 2.7, 95% CI: 1.1–6.5 for group B; HR: 2.5, 95% CI: 1.0–6.0 for group C; HR: 6.2, 95% CI: 2.6–14.7 for group D; HR: 6.5, 95% CI: 2.5–16.7 for group E) comparing with group A. Also, ever smoker had statistically quite higher risk for T2DM (HR: 3.7, 95% CI: 1.2–11.1 for group D; HR: 8.2, 95% CI: 2.5–26.9 for group E). However, there was no interaction (*p* = 0.2280) between WC trajectories and smoking status (i.e., ever smokers, never smokers).

**Table 4:** Hazard ratio with 95% confidence interval for risk of type 2 diabetes according to waist circumference trajectory group by smoking status (*N* = 4, 992)

	Non-smoker (*N* = 3209)			Ever smoker (*N* = 1730)		
	Model1^a^	Model2^b^	Model3^c^	Model1^a^	Model2^b^	Model3^c^
	HR (95% CI)	HR (95% CI)	HR (95% CI)	HR (95% CI)	HR (95% CI)	HR (95% CI)
WC Group A	1.0	1.0	1.0	1.0	1.0	1.0
WC Group B	3.0 (1.3–7.2)	3.0 (1.3–7.2)	2.7 (1.1–6.5)	1.4 (0.5–4.6)	1.4 (0.4–4.6)	1.3 (0.4–4.3)
WC Group C	3.0 (1.2–7.2)	3.0 (1.3–7.3)	2.5 (1.0–6.0)	2.2 (0.7–6.6)	2.2 (0.7–6.6)	1.9 (0.6–5.9)
WC Group D	7.9 (3.4–18.7)	8.2 (3.5–19.4)	6.2 (2.6–14.7)	4.4 (1.5–13.3)	4.4 (1.5–13.1)	3.7 (1.2–11.1)
WC Group E	8.4 (3.3–18.7)	8.8 (3.5–22.3)	6.5 (2.5–16.7)	10.7 (3.3–34.3)	10.5 (1.5–33.8)	8.2 (2.5–26.9)
P for trend	<.0001	<.0001	<.0001	0.0003	<.0001	<.0001
P for interaction	0.2280*					

Abbreviation: *WC* waist circumference, *HR* hazard ratio, *CI* confidence interval; *there was no interaction between WC trajectories and smoking status

^a^Model 1: Adjusted for age, age square, sex and family history of type 2 diabetes mellitus at wave 4

^b^Model 2: Adjusted for Model 2 + alcohol intake, and physical exercise at wave 4

^c^Model 3: Adjusted for Model 3 + systolic blood pressure, and total cholesterol at wave 4

## Discussion

The current study paid particular attention to the application of GBTM for life course data. In our longitudinal population-based cohort study of middle-aged people followed-up biennially for over 14 years, the development of different WC trajectories prior to the diagnosis of T2DM was examined. By using GBTM analysis, five distinct groups of WC change were found among individuals. We could find the majority of individuals as over time increasing abdominal size (Group D and Group E) and they who had high-increasing WC levels over time higher risk for T2DM to 5–7 times. Our analysis revealed different patterns of change in WC as T2DM risk factors considering family history of T2DM, smoking status, alcohol intake, physical exercise, biological indicators of subclinical illness between identified WC trajectories. This finding further highlights that T2DM is a heterogeneous disease with different pathophysiological pathways.

Meanwhile, we could found no gender-interaction, but we additionally confirmed different patterns of WC trend for T2DM risk by using gender-specific trajectory analysis. In men, the model fitting didn’t work well, and the results were also much better in stratification analysis. In other hand, in women, they looked similar to the WC trajectory pattern in entire population, and the results in the gender-specific model were stronger than in stratification analysis. This can be mention to have been no interaction effect when testing interaction effect between WC trajectories and both of gender, as gender-specific results in the gender-specific trajectory are reflected in the entire population model.

In general, the use of BMI as an accurate anthropometric measure in association with T2DM and mortality among adult population has been challenged [22, 23]. Most studies have classified single measured BMI into pre-defined categories. This is currently debatable in relation to mortality [24]. Little research has addressed the heterogeneity or mortality risk of anthropometric measure (i.e. BMI or WC) trajectories in adult populations. Although there were some studies about BMI trajectories, they have not been successful except for studies of children, elderly or whole life cycle [18, 20, 25, 26]. Therefore, in this present study, WC was used as dependent variable to ascertain life-course anthropometric measure trajectory instead of BMI. Instead of studying changes in single measured WC categories, subgroups of WC change over time were defined in this study using latent class trajectory analysis. This kind of statistically progressive method is useful for exploring heterogeneous latent patterns that would not be identified using conventional methods. In addition, the results from the life-course trajectory approach are even better and more efficient in predicting chronic disease risk than traditional approach using single measurement. Indeed, we could identify latent group-specific average patterns of change in WC during follow-up. At this point, we could view about that using a single WC to predict T2DM risk could misclassify risk groups, and the long-term WC changes provide more insight into evolving risk. Recent studies related to life-course approach suggest the cardiometabolic disease consequences of risk factors are cumulative [25]. In this respect, the results of this study are consistent with those of previous studies.

Our latent class trajectory analysis indicated that mean WC levels remained slightly increasing (the “stable weight” group) among specific individuals who developed T2DM during the follow-up. But BMI levels was fairly stable in each group. In our results, groups with increasing WC levels over time had increasing mean values for other clinical variables (i.e., systolic blood pressure, fasting blood sugar) over time also, had higher risk for T2DM than within “stable waist circumference” trajectory groups. However, because they were relatively healthy adults receiving regular physical examinations, their levels were usually within normal clinical ranges. Even the body mass index was very stable within the normal range. This finding suggests that even middle-aged people with normal ranges of factors for metabolic disease may have an increased waist circumference, which may increase the risk of diabetes. Also, there were no flexible WC patterns over time in this study’s results. It is found that anthropometric variables are not changeable in middle-aged, and weight management is important since early adulthood.

In addition, we focused on gender differences, and as our result, show that the relationship between WC and T2DM varies according to gender. Obesity can increase the risk of T2DM in both men and women. Previous studies have shown that the association of T2DM with insulin resistance in normal-weight Chinese is sex-specific. This association is particularly evident in women due to differences in abdominal fat distribution, [11] and there is a tendency for women to have more hepatocellular lipid after glucose and lipid loading compared to men [27, 28]. In this present study, there was no gender interaction by WC trajectory (*P* = 0.1262). In the result of the sensitivity analysis of WC trajectories separately in both of gender to re-confirm differences in WC trajectory patterns between men and women (Additional file 2: Figure S3, and S4), we could found that gender-specific trajectory model did not fit well for men and was shown more strong association with T2DM than before (Additional file 1**:** Table S2, and S3). Therefore, these results from the main analysis and discovery analysis may show concordance with the result of previous study on gender differences in abdominal fat distribution on insight that women have much more hepatocellular lipid than men.

It is well-known that visceral adipose tissue is related to increased cytokine production and insulin resistance (IR) [29]. Also, long-term fatty acid exposure in β-cells can reduce glucose-induced insulin secretion, impair insulin gene expression and increase cell death [30, 31]. However, there is another issue on T2DM pandemic particularly among Asian people. Despite their lower absolute BMI level, they are more prone to visceral fat accumulation and IR than Western populations [32, 33]. They can be called “metabolically obese but normal-weight (MONW)” with normal BMI characterized by a cluster of metabolic risk factors [34]. For MONW, ordinary mechanism for T2DM is not applicable. Instead of that, novel mechanisms such as inherited genetic factor, genetic variation near insulin receptor substrate 1 (IRS1), and ethnic difference in genetics might be involved. Genetic variation near IRS1 is associated with low body fat. It also can impair metabolic profile, including reduced subcutaneous-to-visceral fat ratio, increased insulin resistance, dyslipidemia, and reduced adiponectin level [35].

Many previous studies have shown that weight gain is associated with an increased risk of overweight/obesity mortality and cardiovascular risk [18, 34, 36]. These previous studies also have shown that weight control priorities among middle-aged adults may differ between Korean and Western, and that results on each age groups also differ. Asian’s body fat loads may different compared with Western people and BMI may does not enough reflect differences of body fat loads between Asian and Western [11]. Indeed, in Chinese populations, normal weight obesity with lean shape had significantly higher risks of diabetes, Framingham risk score above 10%, hypertension, and metabolic syndrome than in the normal group [37]. Meanwhile, the findings with WC instead of BMI for trajectory in this study are shown more clear effects of trajectory compared with other BMI trajectories studies, despite of no identifying for clear flexible patterns [20, 23, 27]. Regarding additional result from BMI pattern by each WC trajectory, we could confirm quite stable BMI patterns according to each WC trajectory group (Additional file 2: Figure S1). Also, when BMI trajectory analysis applied to same population of this study, there was no changeable patterns of BMI and clearly significant effects for T2DM risk (not shown result). So, it is the important key point in this study that the association between weight gain and T2DM risk depends on change of abdominal obesity status. Even if BMI level is stable in normal range, high WC level may lead the risk of diabetes to people, but it does not lead those who had stable low WC levels individuals. These results emphasize that BMI is may not a good indicator of the risk of diabetes in the middle-aged population. Therefore, a combination of T2DM risk factors such as WC must be considered. As a result, management of WC through a sophisticated understanding of life expectancy, prognosis assessment and heterogeneity in the progress of risk factors for preventable chronic diseases is important for etiology and prevention.

A major strength of this research lies in its longitudinal data derived from a national representative sample of middle-aged adults over a 14-year period. Using these data in Korea, GBTM analysis was performed first. The association of WC trajectories with risk of T2DM was then examined using cox proportional hazards models. Our results provide important new information regarding trajectories of WC among middle-aged adults and their associations with the risk of diabetes. These results suggest that we can trace WC trajectories to earlier stages of life course. However, it might be important to investigate whether WC trajectories in elderly also display consistent and similar heterogeneity and whether this heterogeneity has similar implications for T2DM risk.

This study has several limitations. First, our approach to model change in WC by time since wave 4 in group-based mixture models failed to distinguish between age effects and period effects [38]. One disadvantage of latent class analysis is that it creates subgroups with very different sizes [39]. However, the latent class growth modeling approach was preferred in this study because it seemed to best capture our interest in describing distinct patterns of change in WC over time among Korean middle aged adults to predict incidences of diabetes. Moreover, in the analysis for the risk of diabetes, cases of T2DM from baseline to wave 4 were excluded and risk factors related to T2DM at entry wave were adjusted for in risk analysis (wave 4). Second, it may be difficult to compare subgroups on the statistical aspect because of different WC trajectory groups. Individuals in the same group tend to be homogeneous, but individual changes from the average of the trajectory group are allowed [11]. Third, in our study, medication data for all WC trajectory groups could not be assessed. This may suggest that overweight and obese individuals who are gaining weight over time are more likely to be screened for T2DM and subsequently receive medication.

## Conclusions

In conclusion, latent class trajectory analysis identified five distinct patterns of WC development prior to a T2DM event. It provides new insights that increasing trajectory is association with high risk of T2DM among the five trajectories identified in this study. This study emphasizes the importance of managing WC across prolong lifespan by assessing the prognosis and prevention strategies of high-risk populations because the WC trajectory of middle-aged adults can be a significant threat to future life expectancy.

## Additional files

Additional file 1: **Table S1.** Differences of mean values from pared t-test according to each WC trajectory group. Abbreviation: SD, standard deviation; SE, standard error; Df, degree of freedom. **Table S2.** Hazard ratio with 95% confidence interval for risk of type 2 diabetes from gender-specific waist circumference trajectory model for men (*N* = 2342). Abbreviation: WC, waist circumference; HR, hazard ratio; CI, confidence interval. ^a^Model 1: Adjusted for age, age square, and family history of type 2 diabetes mellitus at wave 4. ^b^Model 2: Adjusted for Model 2 + smoking status, alcohol intake, physical exercise, and body mass index at wave 4. ^c^Model 3: Adjusted for Model 3 + systolic blood pressure, and total cholesterol at wave 4. **Table S3.** Hazard ratio with 95% confidence interval for risk of type 2 diabetes from gender-specific waist circumference trajectory model for women (*N* = 2650). Abbreviation: WC, waist circumference; HR, hazard ratio; CI, confidence interval. ^a^Model 1: Adjusted for age, age square, and family history of type 2 diabetes mellitus at wave 4. ^b^Model 2: Adjusted for Model 2 + smoking status, alcohol intake, physical exercise, and body mass index at wave 4. ^c^Model 3: Adjusted for Model 3 + systolic blood pressure, and total cholesterol at wave 4. (DOCX 23 kb)

Additional file 2: **Figure S1.** Changes in body mass index during waves 1–4 by trajectory groups of waist circumference. **Figure S2.** Correlation of Trajectories of waist circumference with Body Measurements at wave 4. Abbreviation: BMI, body mass index; WC, waist circumference. *Star marks means that a strong correlation between body mass index and trajectory group of waist circumference was statistically significant (<.0001). **Figure S3.** Trend for waist circumference during waves 1–4 from trajectory analysis in men. **Figure S4.** Trend for waist circumference during waves 1–4 from trajectory analysis in women. (PPTX 207 kb)

## Data Availability

This article is based on data from the Korean Genome and Epidemiology Study (KoGES) conducted by the Korean Centers for Disease Control and Prevention (KCDC). The governmental index of KoGES data are publicly contactable, via http://www.nih.go.kr/NIH/eng/contents/NihEngContentView.jsp?cid=65199&menuIds=HOME004-MNU2261-MNU2262-MNU2263-MNU2264 (last accessed: May 20, 2019). The datasets generated and/or analyzed during the current study are not publicly available as further publications are planned but are available from the corresponding author on reasonable request.

## Abbreviations

BMI: Body mass index
KoGES: Korean Genome and Epidemiology Study
T2DM: Type 2 diabetes mellitus
WC: Waist circumference
