# Supplementary material for: Waist circumference trajectories and risk of type 2 diabetes mellitus in Korean population: the Korean genome and epidemiology study (KoGES)
Source: BMC Public Health. 2019 Jun 13;19:741. doi: 10.1186/s12889-019-7077-6 (PMC6567400; doi:10.1186/s12889-019-7077-6)
Supplement: Supplementary file 1 — Table S1. Differences of mean values from pared t-test according to each WC trajectory group. Abbreviation: SD, standard deviation; SE, standard error; Df, degree of freedom. Table S2. Hazard ratio with 95% confidence interval for risk of type 2 diabetes from gender-specific waist circumference trajectory model for men (N = 2342). Abbreviation: WC, waist circumference; HR, hazard ratio; CI, confidence interval. aModel 1: Adjusted for age, age square, and family history of type 2 diabetes mellitus at wave 4. bModel 2: Adjusted for Model 2 + smoking status, alcohol intake, physical exercise, and body mass index at wave 4. cModel 3: Adjusted for Model 3 + systolic blood pressure, and total cholesterol at wave 4. Table S3. Hazard ratio with 95% confidence interval for risk of type 2 diabetes from gender-specific waist circumference trajectory model for women (N = 2650). Abbreviation: WC, waist circumference; HR, hazard ratio; CI, confidence interval. aModel 1: Adjusted for age, age square, and family history of type 2 diabetes mellitus at wave 4. bModel 2: Adjusted for Model 2 + smoking status, alcohol intake, physical exercise, and body mass index at wave 4. cModel 3: Adjusted for Model 3 + systolic blood pressure, and total cholesterol at wave 4. (DOCX 23 kb) [file 12889_2019_7077_MOESM1_ESM.docx]

**PUBH-D-18-04063R2**

**"Waist circumference trajectories and risk of type 2 diabetes mellitus in Korean population: The Korean Genome and Epidemiology Study (KoGES)"**

Jooeun Jeon^1,2^, Keum Ji Jung^2*^, Sun Ha Jee^2^

^1^Department of Public Health, Graduate School, Yonsei University

^2^Department of Epidemiology and Health Promotion, Institute for Health Promotion, Graduate School of Public Health, Yonsei University

^*^Corresponding author: Keum Ji Jung, Ph. D.

Department of Epidemiology and Health Promotion and Institute for Health Promotion,

Graduate School of Public Health, Yonsei University, 50 Yonsei-ro, Seodaemun-gu, Seoul 03722, Republic of Korea

Tel: 82-2-2228-1541

E-mail: KJJUNG@yuhs.ac

1st author: jjooeun@yuhs.ac

2nd author: jsunha@yuhs.ac

Table S1. Differences of mean values from pared t-test according to each WC trajectory group

|  | No. of Persons | Difference between Wave 1 and Wave 2 | | | | | | Difference between Wave 2 and Wave 3 | | | | | | Difference between Wave 3 and Wave 4 | | | | | |
| --- | --- | --- | --- | --- | --- | --- | --- | --- | --- | --- | --- | --- | --- | --- | --- | --- | --- | --- | --- |
|  |  | Mean | SD | S.E. mean | Paired t-test | | | Mean | SD | S.E. mean | Paired t-test | | | Mean | SD | S.E. mean | Paired t-test | | |
|  |  |  |  |  | t value | Df | p-value |  |  |  | t value | Df | p-value |  |  |  | t value | Df | p-value |
| Group A  (Very low-stable) | 635 | -0.6 | 4.7 | 0.2 | -3.3 | 634 | 0.0009 | 1.4 | 3.6 | 0.1 | 10.2 | 634 | <.0001 | -1.2 | 4.2 | 0.2 | -7.1 | 634 | <.0001 |
| Group B  (Low-stable) | 1426 | 0.3 | 5.0 | 0.1 | 2.0 | 1425 | 0.0450 | 1.2 | 4.0 | 0.1 | 10.9 | 1425 | <.0001 | -0.7 | 4.6 | 0.1 | -5.8 | 1425 | <.0001 |
| Group C  (Moderate-stable) | 1662 | 0.9 | 4.6 | 0.1 | 7.8 | 1661 | <.0001 | 0.7 | 3.9 | 0.1 | 7.8 | 1661 | <.0001 | 0.2 | 4.4 | 0.1 | 2.3 | 1661 | 0.0567 |
| Group D  (Elevated-increasing) | 1012 | 1.8 | 4.5 | 0.1 | 13.1 | 1011 | <.0001 | 0.4 | 4.2 | 0.1 | 2.9 | 1011 | <.0001 | 0.9 | 4.6 | 0.1 | 6.6 | 1011 | <.0001 |
| Group E  (High-increasing) | 257 | 2.7 | 4.8 | 0.3 | 8.9 | 256 | <.0001 | 3.3 | 4.6 | 0.3 | 1.1 | 256 | 0.0045 | 0.6 | 4.8 | 0.3 | 1.9 | 256 | 0.0226 |

Abbreviation: SD, standard deviation; SE, standard error; Df, degree of freedom

Table S2. Hazard ratio with 95% confidence interval for risk of type 2 diabetes from gender-specific waist circumference trajectory model for men (N=2,342)

|  |  | *No. of Persons* | *No. of T2DM incidences* | *Model1^a^* | *Model2^b^* | *Model3^c^* | *Model 4^d^* |
| --- | --- | --- | --- | --- | --- | --- | --- |
|  |  |  |  | HR (95% CI) | HR (95% CI) | HR (95% CI) | HR (95% CI) |
| *WC Group A* | | 196 | 5 | 1.0 | 1.0 | 1.0 | 1.0 |
| *WC Group B* | | 612 | 21 | 1.3 (0.5-3.5) | 1.3 (0.5-3.4) | 1.2 (0.4-3.2) | 1.1 (0.4-3.1) |
| *WC Group C* | | 859 | 39 | 1.7 (0.7-4.3) | 1.7 (0.7-4.3) | 1.5 (0.6-3.9) | 1.4 (0.5-3.9) |
| *WC Group D* | | 519 | 48 | **3.5 (1.4-8.9)** | **3.5 (1.4-8.8)** | **2.9 (1.2-7.4)** | 2.5 (0.8-7.8) |
| *WC Group E* | | 156 | 29 | **7.6 (2.9-19.6)** | **7.5 (2.9-19.3)** | **6.1 (2.4-16.0)** | **5.0 (1.3-18.8)** |
| *P for trend* | |  |  | <.0001 | <.0001 | <.0001 | 0.0009 |

Abbreviation: WC, waist circumference; HR, hazard ratio; CI, confidence interval

^a^Model 1: Adjusted for age, age square, and family history of type 2 diabetes mellitus at wave 4

^b^Model 2: Adjusted for Model 2 + smoking status, alcohol intake, physical exercise, and body mass index at wave 4

^c^Model 3: Adjusted for Model 3 + systolic blood pressure, and total cholesterol at wave 4

Table S3. Hazard ratio with 95% confidence interval for risk of type 2 diabetes from gender-specific waist circumference trajectory model for women (N=2,650)

|  |  | *No. of Persons* | *No. of T2DM incidences* | *Model1^a^* | *Model2^b^* | *Model3^c^* | *Model 4^d^* |
| --- | --- | --- | --- | --- | --- | --- | --- |
|  |  |  |  | HR (95% CI) | HR (95% CI) | HR (95% CI) | HR (95% CI) |
| *WC Group A* | | 422 | 4 | 1.0 | 1.0 | 1.0 | 1.0 |
| *WC Group B* | | 817 | 31 | **3.9 (1.4-11.0)** | **3.9 (1.4-10.9)** | **3.6 (1.3-10.1)** | **3.0 (1.0-8.6)** |
| *WC Group C* | | 758 | 34 | **4.3 (1.5-12.2)** | **4.4 (1.5-12.5)** | **3.7 (1.3-10.5)** | 2.7 (0.9-8.1) |
| *WC Group D* | | 512 | 48 | **9.0 (3.2-25.5)** | **9.3 (3.3-26.5)** | **7.1 (2.5-20.2)** | **4.4 (1.4-14.1)** |
| *WC Group E* | | 141 | 17 | **11.6 (3.8-35.3)** | **12.1 (4.0-36.7)** | **8.8 (2.8-27.1)** | **4.1 (1.0-16.7)** |
| *P for trend* | |  |  | <.0001 | <.0001 | <.0001 | 0.0501 |

Abbreviation: WC, waist circumference; HR, hazard ratio; CI, confidence interval

^a^Model 1: Adjusted for age, age square, and family history of type 2 diabetes mellitus at wave 4

^b^Model 2: Adjusted for Model 2 + smoking status, alcohol intake, physical exercise, and body mass index at wave 4

^c^Model 3: Adjusted for Model 3 + systolic blood pressure, and total cholesterol at wave 4
