# Supplementary material for: Waist circumference trajectories and risk of type 2 diabetes mellitus in Korean population: the Korean genome and epidemiology study (KoGES)
Source: BMC Public Health. 2019 Jun 13;19:741. doi: 10.1186/s12889-019-7077-6 (PMC6567400; doi:10.1186/s12889-019-7077-6)
Supplement: Supplementary file 2 — Figure S1. Changes in body mass index during waves 1–4 by trajectory groups of waist circumference. Figure S2. Correlation of Trajectories of waist circumference with Body Measurements at wave 4. Abbreviation: BMI, body mass index; WC, waist circumference. *Star marks means that a strong correlation between body mass index and trajectory group of waist circumference was statistically significant (<.0001). Figure S3. Trend for waist circumference during waves 1–4 from trajectory analysis in men. Figure S4. Trend for waist circumference during waves 1–4 from trajectory analysis in women. (PPTX 207 kb) [file 12889_2019_7077_MOESM2_ESM.pptx]

## Slide 1
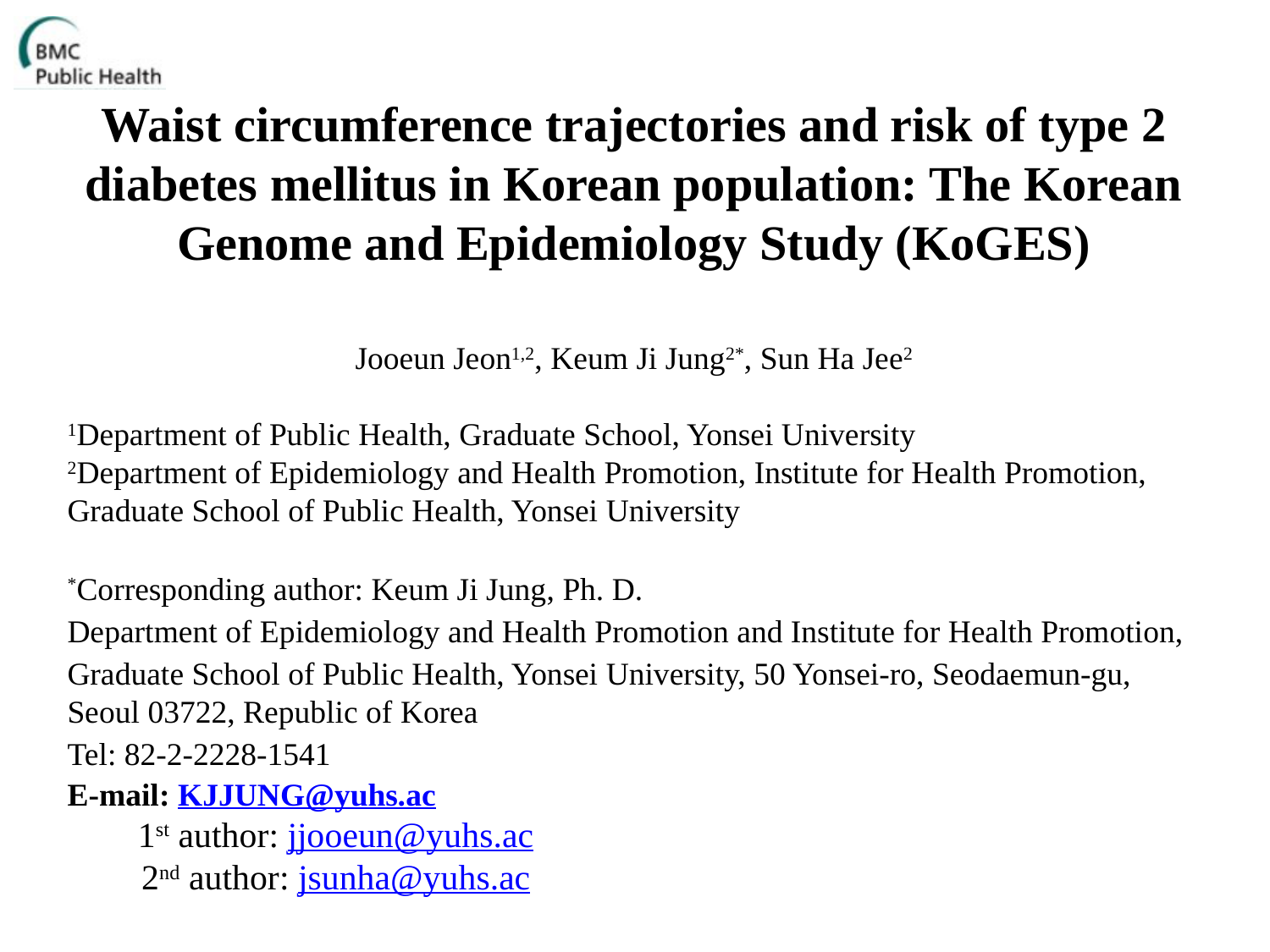

Waist circumference trajectories and risk of type 2 diabetes mellitus in Korean population: The Korean Genome and Epidemiology Study (KoGES)
Jooeun Jeon1,2, Keum Ji Jung2*, Sun Ha Jee2
1Department of Public Health, Graduate School, Yonsei University
2Department of Epidemiology and Health Promotion, Institute for Health Promotion,
Graduate School of Public Health, Yonsei University
*Corresponding author: Keum Ji Jung, Ph. D.
Department of Epidemiology and Health Promotion and Institute for Health Promotion,
Graduate School of Public Health, Yonsei University, 50 Yonsei-ro, Seodaemun-gu, Seoul 03722, Republic of Korea
Tel: 82-2-2228-1541
E-mail: KJJUNG@yuhs.ac
1st author: jjooeun@yuhs.ac
2nd author: jsunha@yuhs.ac

## Slide 2
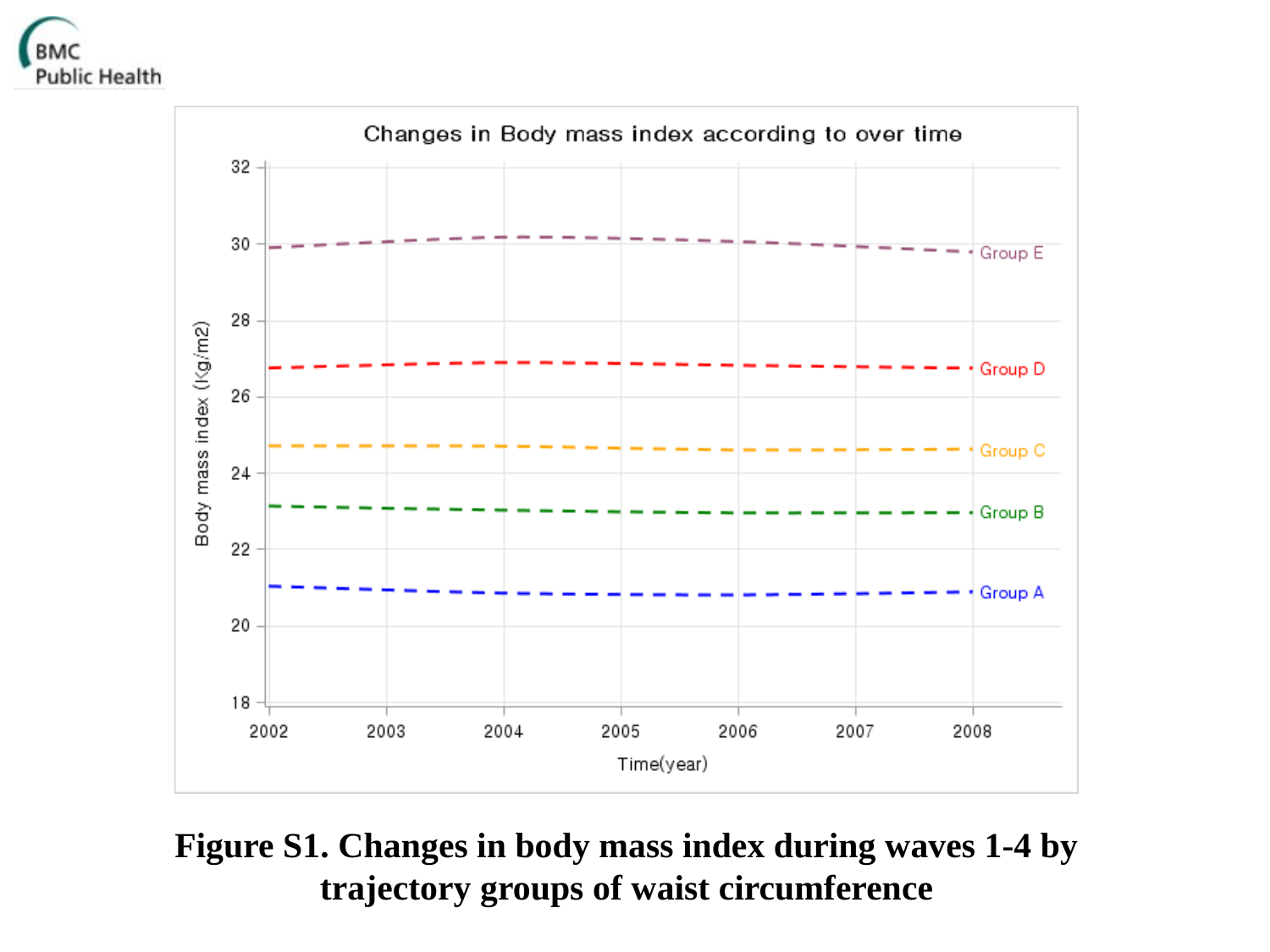

Figure S1. Changes in body mass index during waves 1-4 by trajectory groups of waist circumference

## Slide 3
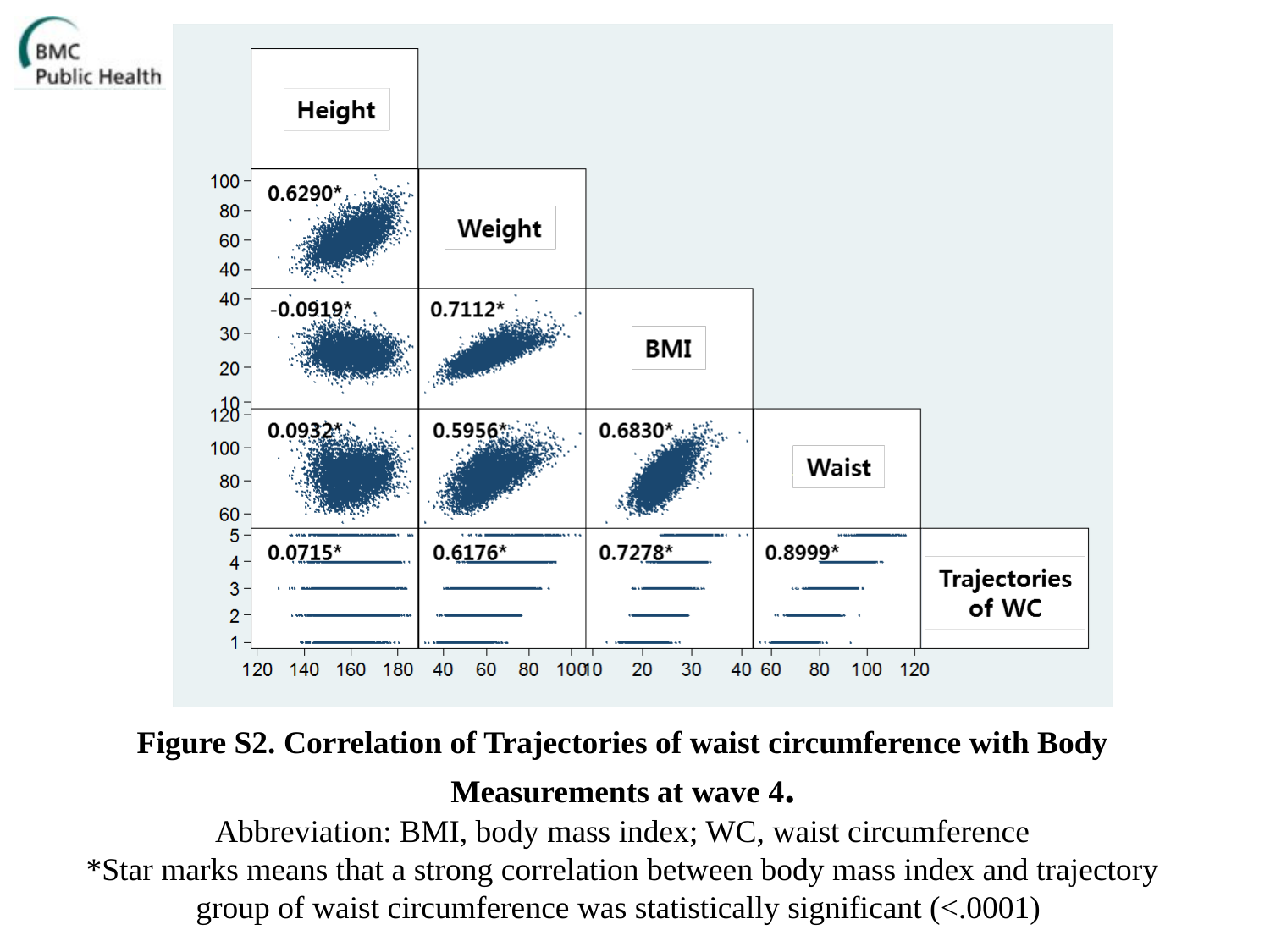

Figure S2. Correlation of Trajectories of waist circumference with Body Measurements at wave 4.
Abbreviation: BMI, body mass index; WC, waist circumference
*Star marks means that a strong correlation between body mass index and trajectory group of waist circumference was statistically significant (<.0001)

## Slide 4
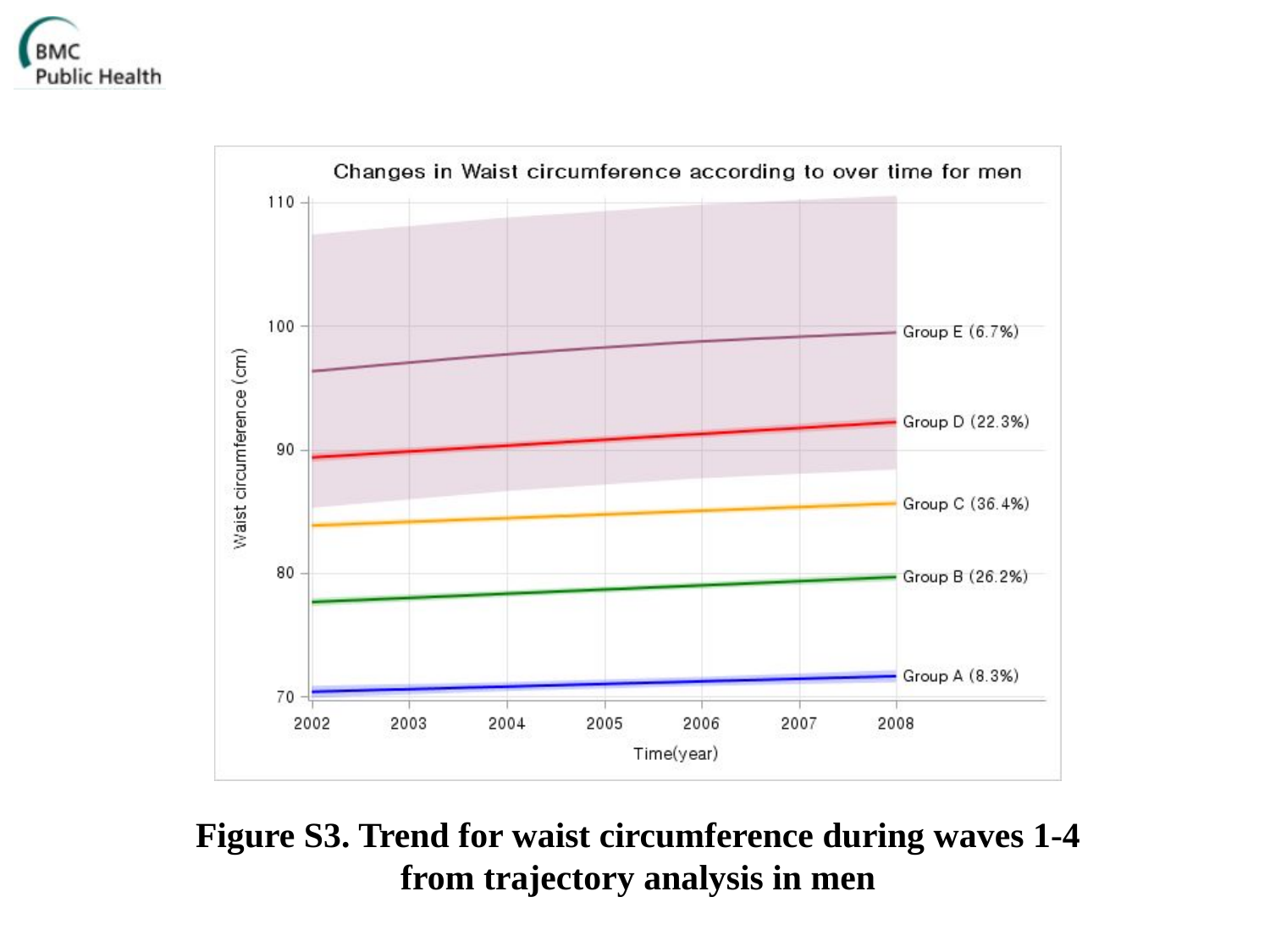

Figure S3. Trend for waist circumference during waves 1-4 from trajectory analysis in men

## Slide 5
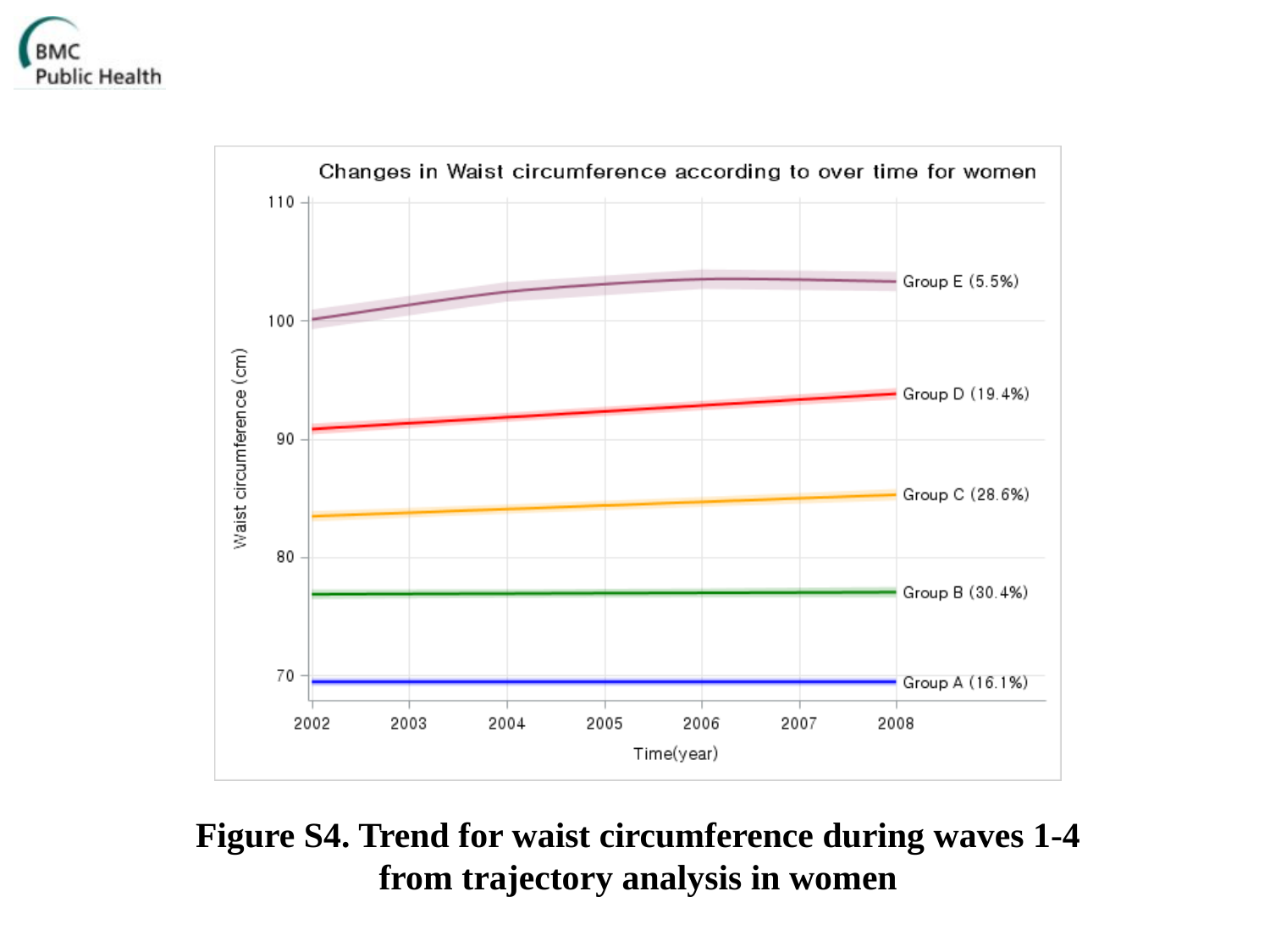

Figure S4. Trend for waist circumference during waves 1-4 from trajectory analysis in women
